# Alveolar Differentiation Potency of Human Distal Airway Stem Cells Is Associated with Pulmonary Pathological Conditions

**DOI:** 10.1155/2019/7123078

**Published:** 2019-06-09

**Authors:** Yujia Wang, Yi Lu, Yingchuan Wu, Yufen Sun, Yueqing Zhou, Qiwang Ma, Yawen Zheng, Qianwen Yu, Yujie Cao, Guangdong Chen, Ting Zhang, Xiaotian Dai, Tao Ren, Yu Ma, Wei Zuo

**Affiliations:** ^1^East Hospital, School of Medicine, Tongji University, Shanghai 200120, China; ^2^Kiangnan Stem Cell Institute, Zhejiang 311300, China; ^3^Shanghai Jiao Tong University Affiliated Sixth People's Hospital, Shanghai 200233, China; ^4^Southwest Hospital, Third Military Medical University of PLA, Chongqing 400038, China; ^5^Ningxia Medical University, Yinchuan 750004, China

## Abstract

**Background:**

This study is aimed at characterizing the human distal airway stem cells (DASCs) and assessing their therapeutic potential in patients with chronic, degenerative lung diseases. These findings will provide a comprehensive understanding for further clinical applications utilizing autologous airway stem cells as therapeutic intervention in respiratory diseases.

**Methods:**

DASCs were isolated from healthy subjects or patients diagnosed with bronchiectasis, chronic obstructive pulmonary diseases (COPD), or interstitial lung disease (ILD). Differentiation capacity, a key property of the stem cells, was studied using a novel monolayer differentiation system. The differentiated cells were evaluated for alveolar and bronchial cell marker expression, and the quantified expression level of differentiated cells was further examined for their relationship with age and pulmonary function of the subjects.

**Results and Conclusions:**

Differentiation of DASCs and tracheal stem cells (TSCs) yielded an alveolus-like structure and a tube-shaped structure, respectively, with distinct marker gene expression. Additionally, single-cell-derived clones showed diverse differentiation fates, even if the clones arise from identical or different individuals. More importantly, the alveolar differentiation potency was higher in DASCs derived from patients than from healthy people. The differentiation efficiency of DASCs also correlates with age in patients with bronchiectasis and ILD.

## 1. Introduction

In the modern world, lung disease is one of the major threats of public health worldwide, with high morbidity and mortality surpassed only by cardiovascular disease and cancer. For instance, some population-based studies emphasized that the overall prevalence of COPD ranged from 7.8% to 19.7% around the world, accounting for more than 700 million people with COPD worldwide [1–6]. While lung transplantation serves as the ultimate and most effective medical option for end-stage lung disease, shortage of donor organ and chronic rejection posttransplantation are limiting the long-term survival of recipients [7]. Cell therapy using tissue-specific multipotential stem cells holds great potential as a novel strategy for lung diseases characterized by irreversible, progressive damage of airway and alveolar tissues, such as bronchiectasis, idiopathic pulmonary fibrosis (IPF), and chronic obstructive pulmonary disease (COPD).

Previous researches conducted by our group and others have identified local populations of adult lung stem/progenitor cells in airway and alveolar tissues that respond to injury and airway epithelia regeneration [8–15]. They are capable of undergoing long-term self-renewal and give rise to multiple differentiated cell types. In our previous work, we reported that a cell subtype existing in mouse and human distal airway basement membrane can be cloned and propagated as single-cell-derived pedigrees in vitro [9, 16]. This migratory basal cell population has been proven to assist lung regeneration after severe H1N1 influenza infection in mice [8]. More importantly, this human SOX9+P63+KRT5+ distal airway stem cell (DASC) population has been evaluated as a potent candidate for cell-based therapy for lung disease. From a trace amount of airway epithelium collected by bronchoscopy, the isolated DASCs can expand by orders of magnitude in vitro, showing regenerative capacity with both human bronchiolar and alveolar epithelia reconstituted when transplanted into injured immune-deficient mouse lungs. Meanwhile, a clinical trial enrolling two patients diagnosed with noncystic fibrosis bronchiectasis revealed restoration of the airway structure and improvement of pulmonary function after autologous-cell intratracheal transplantation [16].

Although great progress has been achieved, full characterization of the lung stem/progenitor cells under homeostatic and pathological conditions remains to be addressed. There are a number of reports detailing stem cell alteration after being continuously exposed to endogenous or environmental stimulus, including cigarette intake and inflammation. Ghosh et al. and Shaykhiev reported a depletion and dysregulation of airway basal progenitors in smokers with COPD [17, 18]. Another research on a ferret lung transplant model of obliterative bronchiolitis showed that basal cell proliferative capacity declines with progression of disease and phenotypic changes [19]. To gain further insights into the behavior of the DASCs isolated from a patient with lung diseases, we analyzed their differentiation capacity in a monolayer, serum-free culture condition. Whether age and disease would play a role in DASC differentiation potential was assessed. The result in the present study will provide a basis for further clinical trials utilizing autologous lung stem/progenitor cells as therapeutic intervention in respiratory diseases.

## 2. Materials and Methods

### 2.1. Study Approval

This was a cross-sectional clinical study (ClinicalTrials.gov identifier: NCT03153800) to analyze the differentiation capacity of DASC in healthy subjects and patients diagnosed with bronchiectasis, ILD, or COPD. The trial was approved by the ethics committee at each participating institution and conducted in compliance with the Good Clinical Practice (GCP) standard and the Declaration of Helsinki. All subjects were informed in detail of the objective and study design of the current study, and signed informed consents were obtained.

### 2.2. Participants

Six healthy volunteers with normal spirometry and 13 patients with bronchiectasis, 21 patients with COPD, and 45 patients with ILD were recruited by their chest physician from 5 hospitals. Diagnosis was established according to ATS/ERS guidelines. Inclusion criteria for the study were as follows: diagnosed with bronchiectasis, COPD, or ILD according to the guideline, clinically stable for more than 4 weeks, and tolerant to fiberoptic bronchoscopy. Exclusion criteria for the study were as follows: malignant tumor, syphilis, HIV, severe pulmonary infection, undergoing active treatment for anti-infection, severe heart disease, a history of abusing alcohol and illicit drug, participated in other clinical trials in the past 3 months, pregnant or lactating, or assessed as inappropriate to participate in the current study by investigators. For each enrolled participant, personal information including age, gender, body mass index, and medical history was recorded. Medical examination based on standardized clinical SOPs was performed 24 hours before bronchoscopy.

### 2.3. Airway Epithelium Sampling

The bronchoscopic procedure for lung epithelial sampling was performed by respiratory physicians using a flexible fiberoptic bronchoscope (Olympus, Japan) [16]. Briefly, oropharyngeal anesthesia and laryngeal anesthesia were achieved by administration of 2 mL nebulized 4% lidocaine, followed by 1 mL 2% topical lidocaine sprayed into the oral and nasal cavities. After the bronchoscope was advanced through the vocal cords, 2 mL 2% lidocaine solution was instilled into the trachea and both main bronchi through the bronchoscope. A disposable 2 mm brush was advanced through the working channel of the bronchoscope and used to collect airway epithelial cells by gently brushing back and forth 1-2 times in random regions of the trachea or 4^th^-order bronchi. Trachea samples were used to generate TSC clones, and samples from the 4^th^-order bronchi were used to generate DASC clones.

### 2.4. DASC and TSC Isolation and Expansion

The bronchoscopic brush with the trachea or bronchus epithelium sample was cut into 1 cm long pieces. After removing the sputum, the brush pieces were directly immerged into dissociation buffer (DMEM/F12 medium with 2 mg/mL protease XIV, 0.01% trypsin, and 10 ng/mL DNase I) and incubated for 1 hour at 37°C with gentle shaking. After dissociation, cell samples were passed through a 70 *μ*m nylon mesh (Falcon, USA) to remove aggregates and washed three times with ice-cold F12 medium supplemented with 5% FBS and 1% Pen/Strep. Viable cell counts were determined using a hemocytometer. Cell pellets were collected by centrifugation at 200 × g and directly plated onto mitomycin-inactivated 3T3 fibroblast feeder cells and cultured under 7.5% CO_2_ condition as previously described [9]. To obtain a single-cell-derived clone, a clone cylinder (Sigma, USA) and high vacuum grease (Dow Corning, USA) were used to pick up a single colony grown up from one cell. Typically, both DASC and TSC are passaged every 4-6 days in a 1 : 7 ratio.

### 2.5. Monolayer Differentiation of DASC and TSC In Vitro

After removing feeders by differential trypsinization, DASCs or TSCs were plated on 12-well plates (10^4^ cells per well) precoated with 15% cold collagen type I (Corning, USA) and 20% Matrigel (Corning, USA). 24 hours after seeding, the culture medium was changed to differentiation medium (DMEM/F12 supplemented with 1% Pen/Strep, 5 mg/mL insulin, 5 mg/mL transferrin, 0.025 mg/mL cholera toxin, 5 ng/mL EGF, 30 mg/mL BPE, 1 mg/mL BSA, 50 ng/mL FGF10, and 30 ng/mL HGF) for 5-12 days. FGF10 and HGF were used to favor distal lung fate [20–22]. Airway differentiation was assessed by expression of key markers of mature alveolar and bronchial cells.

### 2.6. Immunofluorescence Experiments

Cell culture was fixed and stained using standard protocols which have been described previously [16]. Antibodies used in the current study were anti-Krt5 (1 : 200, EP1601Y, Thermo Fisher Scientific, USA), anti-p63 (1 : 500, 4A4, Abcam, USA), anti-Krt14 (1 : 500, EPR17350, Abcam, USA), HuNu (1 : 200, 235-1, Abcam, USA), Ki67 (1 : 500, B126.1, Abcam, USA), AQP5 (1 : 500, EPR3747, Abcam, USA), HOPX (1 : 200, ab230544, Abcam, USA), PDPN (1 : 200, 18H5, Santa Cruz Biotechnology, USA), CC10 (1 : 200, T-18, Santa Cruz Biotechnology, USA), and LAMP3 (1 : 200, 12632-1-AP, Proteintech, USA). Alexa Fluor-conjugated 488/594 (1 : 200, Life Technologies, USA) antibodies were used as secondary antibodies.

### 2.7. Quantitative PCR

Total RNA was isolated following the manufacturer's instructions (RNeasy Mini Kit, QIAGEN). 1 *μ*g total RNA was transcribed into cDNA (PrimeScript™ cDNA Synthesis Kit, Takara). Gene expression was analyzed using SYBR® Premix Ex Taq™ II (Takara) and an ABI 7500 real-time PCR system (Applied Biosystems, USA). Each biological sample was analyzed in experimental replicates (*n* = 3 repeated wells) with the Ct value of each replicate being averaged and was normalized to reference gene GAPDH. Fold change was calculated by the 2^−ΔΔCt^ method.

### 2.8. FACS Analysis

Cells were trypsinized into a single cell suspension, fixed by 3.7% paraformaldehyde for 15 min, and permeabilized by 0.2% Triton X-100 for 5 min. All samples were blocked in 1% donkey serum for 30 minutes, followed by sequential incubation with primary antibody and FITC/APC-Cy7-conjugated secondary antibody (1 : 400, Life Technologies, USA). The single cell suspension was prepared using a 30 *μ*m preseparation filter (Miltenyi Biotec, Germany) before the test. A BD FACSVerse (BD, USA) equipped with 488 and 647 lasers was used to detect the fluorescence signals. FSC-A and SSC-A parameters were used to exclude the debris, and FSC-H, FSC-W, and SSC-W parameters were used to exclude clusters in the cell suspension. Nonimmune IgG control samples were used to set the bottom line of the positive signals.

### 2.9. Quantification of Alveolar Differentiation Potency

A differentiated cell suspension harvested on day 5 was used for immunofluorescent staining to determine the alveolar differentiation potency of DASCs sampled from each subject. Briefly, cells were fixed by 3.7% paraformaldehyde and permeabilized by 0.2% Triton X-100. After incubation with 5% donkey serum for 1 hour, cells were stained with HOPX primary antibody or IgG control at 4°C overnight followed by incubation with Alexa Fluor-conjugated 594 secondary antibody for 2 hours. An image-based cell counter (Countess II FL, Thermo Fisher Scientific, USA) with a fluorescent filter set for Alexa Fluor-conjugated 594 was used to test the HOPX+ cell frequency. The IgG control sample was used to set the bottom line of the fluorescent signals. Gating parameters to include target cells or exclude debris were cell size, brightness, circularity, and fluorescence intensity. Similar gating strategies were used to ensure the consistency of the measurement.

### 2.10. Statistical Analyses

All statistical analyses were performed using GraphPad Prism (GraphPad Software Inc., USA). For all experiments, the *n* value represents an independent biological sample. For normally distributed data sets, differences were evaluated using one-way ANOVA and post hoc analysis was performed using the Tukey-Kramer Multiple Comparison Test when ANOVA indicated significance. For nonnormally distributed data sets, a two-tailed Mann–Whitney *U* test was used to evaluate the differences. Two-tailed Pearson correlations were used for all correlation analyses. All statistical significance is reported accordingly. ^∗^ *p* < 0.05, ^∗∗^ *p* < 0.01, and ^∗∗∗^ *p* < 0.001. Results are represented as mean ± standard deviation.

## 3. Results

### 3.1. Clonogenicity and In Vitro Differentiation Capacity of Human DASCs

**Figure 1:**
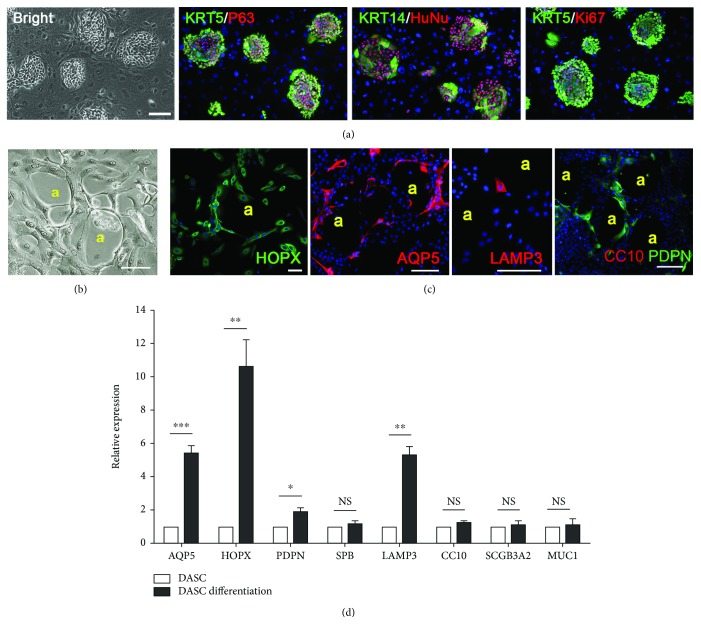
Characterization of human DASC culture in vitro. (a) Human DASC colonies immunostained with KRT5, P63, KRT14, HuNu, and Ki67. Nuclei were labeled with DAPI (blue). Scale bar, 100 *μ*m. (b) Representative images of monolayer-differentiated DASCs forming an alveolus-like structure on day 12. (A) Alveolus-like structure. Scale bar, 50 *μ*m. (c) Immunostaining of indicated AEC1 markers (HOPX, AQP5, and PDPN), AEC2 marker (LAMP3), and bronchiolar marker (CC10) on monolayer-differentiated DASCs. (A) Alveolus-like structure. Scale bar, 50 *μ*m. (d) qPCR showing alveolar and bronchial epithelium marker gene expression of DASCs before and after differentiation. ^∗^ *p* < 0.05, ^∗∗^ *p* < 0.01, and ^∗∗∗^ *p* < 0.001. NS: not significant.

Human airway stem cells derived from the 4^th^-order bronchi can be successfully cloned and propagated on a feeder culture system in vitro, expressing lung epithelial stem cell markers cytokeratin 5 (KRT5) and transformation protein 63 (P63), as well as the human-specific marker human nuclear antigen (HuNu) [10]. Clones were composed of KRT5+P63+KRT14+ and KRT5+P63+KRT14- subpopulations [23]. The Ki67 expression of proliferating cells in the clone revealed robust cell proliferation of the isolated human cells (Figure 1(a)). Consistent with our previous work, 3.83 ± 1.41 cell clones arise from the cell suspension containing around 2000 brushed cells. The clone number, DASC morphology, and key marker expression were assessed among samples isolated from the 4^th^-order bronchi or different lobes of the lung; however, no differences were observed (data not shown).

Here, we introduced a novel monolayer differentiation system to study the alveolar differentiation capacity of human DASCs. When subjected to the differentiation, the feeder-free DASC culture gave rise to a few alveolus-like structures lined by thin, highly elongated cells (Figure 1(b)). Immunostaining showed that most cells lining the alveolus-like structures expressed HOPX, AQP5, and PDPN, which are known markers of type I alveolar epithelial cell (AEC1). Expression of type II alveolar epithelial cell (AEC2) marker LAMP3 was also observed in a small portion of differentiated cells. PDPN, but not club cell marker CC10 (also known as SCGB1A1), expression was also detected in such structures (Figure 1(c)). A consistent result was obtained by qPCR analysis (Figure 1(d)). The above data indicated that the KRT5+P63+ population derived from human bronchi has a unique potential of differentiating to alveolar lineage including AEC1 and AEC2.

### 3.2. Clonogenicity and In Vitro Differentiation Capacity of Human TSCs

**Figure 2:**
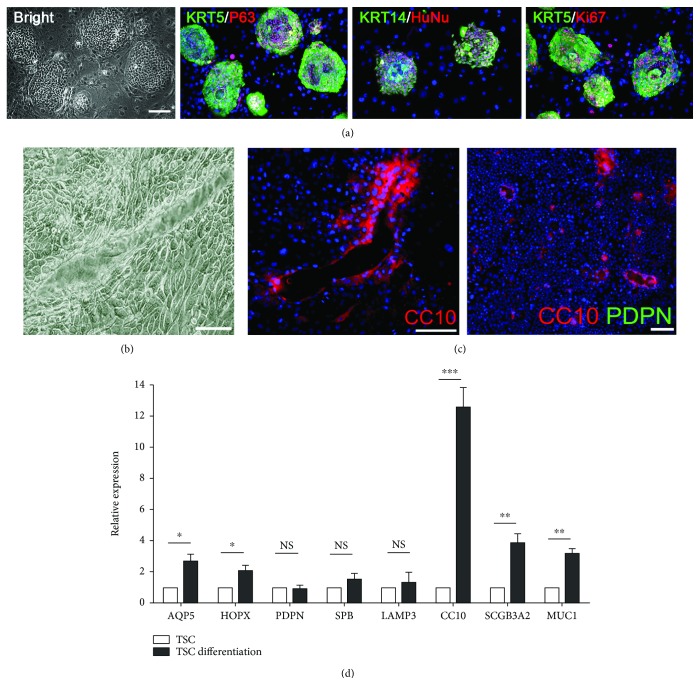
Characterization of human TSC culture in vitro. (a) Human TSC colonies immunostained with KRT5, P63, KRT14, HuNu, and Ki67. Nuclei were labeled with DAPI (blue). Scale bar, 100 *μ*m. (b) Representative images of monolayer-differentiated TSCs form the structure with tube-shaped cavity on day 12. Scale bar, 100 *μ*m. (c) Immunostaining of indicated alveolar (PDPN) and bronchial (CC10) epithelium markers on monolayer-differentiated TSCs. Scale bar, 50 *μ*m. (d) qPCR showing alveolar and bronchial epithelium marker gene expression of TSCs before and after differentiation. ^∗^ *p* < 0.05, ^∗∗^ *p* < 0.01, and ^∗∗∗^ *p* < 0.001. NS: not significant.

Besides KRT5+P63+ DASCs, tracheal stem cells (TSCs) existing in the proximal lung also exhibited enormous regenerative potential in the injured lung [24]. Here, we isolated and expanded the clonogenic human TSCs from the trachea using the same minimally invasive bronchoscopic brushing strategy. As expected, human TSC colonies express KRT5 and P63 and showed morphology indistinguishable from human DASCs (Figure 2(a)).

When subjected to the same 2D monolayer differentiation system, TSCs gave rise to structures with a tube-shaped cavity, which is distinct from the alveolus-like structure formed by DASCs (Figure 2(b)). Moreover, TSC-derived structures did not express any alveolar marker but expressed differentiated club cell markers, as assayed by immunofluorescence (Figure 2(c)) and qPCR (Figure 2(d)). Altogether, the data indicated that clonogenic DASCs and TSCs isolated from different anatomic regions of the human airway demonstrated distinct lineage commitment.

### 3.3. Lineage Diversity of DASCs at Single Cell Resolution

**Figure 3:**
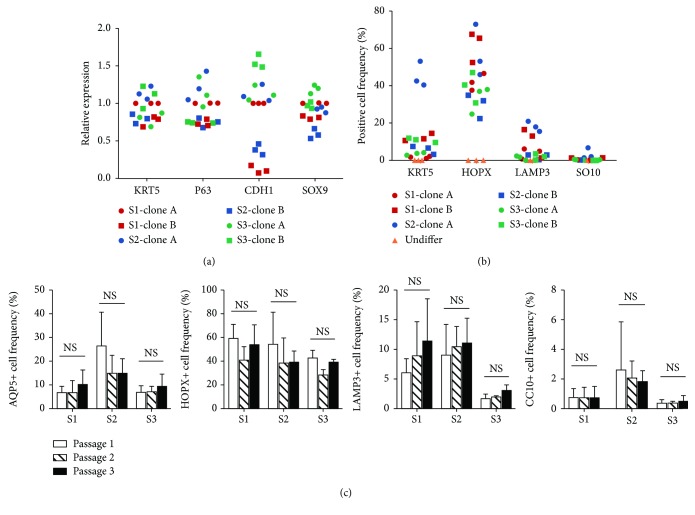
Diversity analysis of DASC differentiation potency at single cell resolution. (a) qPCR analysis of stem cell markers KRT5, P63, CDH1, and SOX9 expressed in 6 different single-cell-derived DASC clones originated from 3 individuals. 2 clones (clone A and clone B) were generated from each individual. (b) Diagram showing the ratio of AQP5+, HOPX+, LAM3+, and CC10+ cells generated from different single-cell-derived clones after 12 days of monolayer differentiation. DASCs before monolayer differentiation (Undiffer) were used as a negative control. (c) AQP5+, HOPX+, LAM3+, and CC10+ cell frequencies in differentiated DASCs at different culture passages. S1: healthy subject; S2: subject with ILD; S3: subject with bronchiectasis.

The long-term self-renewal ability, one of the most prominent features of DASCs, has allowed isolation of independent pedigrees which were composed entirely of the progeny from a single cell [8]. We next sought to determine whether single-cell-derived pedigrees from different individuals were endowed with different capacities upon differentiation. We performed the differentiation assay on 6 distinct single-cell-derived DASC clones originated from 3 subjects (S1: healthy subject, S2: subject with ILD, and S3: subject with bronchiectasis.). Before differentiation, mRNA levels of epithelial stem cell markers including KRT5, P63, CDH1, and SOX9 were determined by qPCR, showing uniform expression in KRT5, P63, and SOX9, but not CDH1 (Figure 3(a)). After differentiation, cells were characterized by flow cytometry using AQP5, HOPX, LAMP3, and CC10 markers (Figure 3(b)). The data indicated that independent single-cell-derived clones from the same subject may have diverse differentiation potential. Differentiation diversity was further assessed by comparing the differentiation capacity of DASCs at different passages. Consequently, no significant difference among DASCs from passage 1, passage 2, and passage 3 was observed (Figure 3(c)). Taken together, these data suggested that the lineage diversity of DASC is mainly attributed to the differentiation bias of single cells, but not different culture passages.

### 3.4. Different Differentiation Potencies of DASCs from Normal and Diseased Lungs

**Figure 4:**
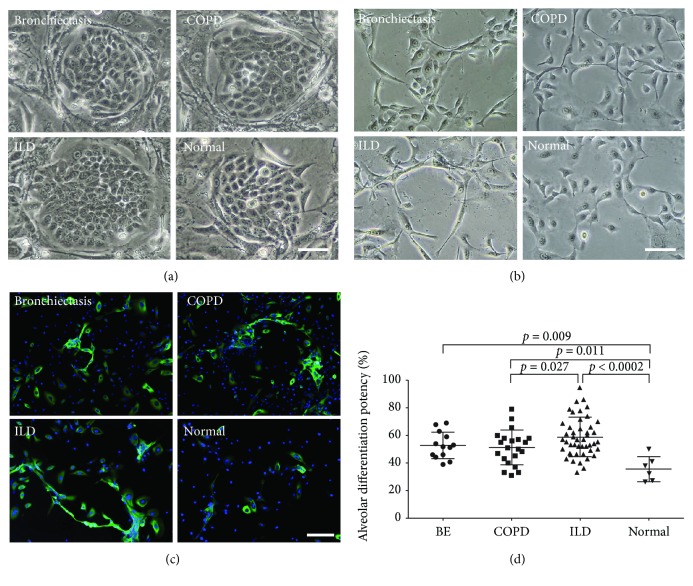
DASC phenotype from normal and diseased lungs. (a) Representative cell culture images of DASCs generated from bronchiectasis, COPD, ILD, and normal lungs. Scale bar, 50 *μ*m. (b) Representative images of monolayer-differentiated DASCs from bronchiectasis, COPD, ILD, and normal lungs. Scale bar, 50 *μ*m. (c) Immunostaining of HOPX expression on monolayer-differentiated DASCs from bronchiectasis, COPD, ILD, and normal lungs. Scale bar, 50 *μ*m. (d) Quantification of alveolar differentiation potency of DASCs generated from bronchiectasis, COPD, ILD, and normal lungs. Horizontal lines represent the mean within groups, with standard deviation error bars. Normal refers to healthy volunteers with normal spirometry.

We expanded the differentiation assay to a much larger cohort, aiming to figure out whether DASCs under pathophysiological conditions are able to recapitulate a potent differentiation fate in patients with chronic degenerative lung diseases. Bronchoscopic samples were collected and DASCs were isolated from 85 subjects (6 healthy volunteers, 13 patients with bronchiectasis, 21 patients with COPD, and 45 patients with ILD). The baseline patient characteristics are shown in Table 1. Interestingly, DASC clones endowed with similar clonogenicity can be yielded from both normal and diseased lungs (Figure 4(a)). Next, alveolar differentiation potency was quantified by immunofluorescence staining and a fluorescent quantitative analysis (Figure 4(b)). HOPX+ cell frequency was set as a parameter to determine the differentiation potency of the DASCs from all subjects because the HOPX expression can be detected as early as day 5 during differentiation and its expression pattern is similar to those of AQP5 and PDPN (Figure 4(c) and Supplementary Figure 1). Interestingly, we found that the differentiated AEC1 number was significantly increased in the bronchiectasis, COPD, and ILD groups compared to healthy subjects (Figure 4(d)). These results indicated that although DASC clones from normal and diseased lungs exhibited similar phenotypes, their intrinsic differentiation capacity may be enhanced under pathophysiological conditions.

### 3.5. Correlation of DASC Differentiation Potency with Gender, Age, and Lung Function

**Figure 5:**
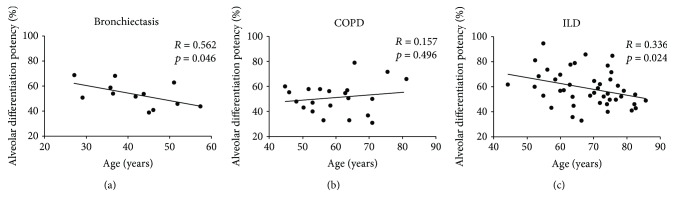
The correlation between age and alveolar differentiation potency of DASCs. The DASCs from bronchiectasis, COPD, and ILD patients were examined by Pearson correlations to determine whether alveolar differentiation potency was related to the age of the patients.

Correlations of alveolar differentiation potency with age and gender of the subjects with different lung diseases were also performed. While gender did not correlate with DASC differentiation, tight relationships between age and alveolar differentiation potency were observed in bronchiectasis (*r* = −0.562 and *p* = 0.046) and ILD (*r* = −0.336 and *p* = 0.024) patients, but not in COPD patients (*r* = 0.157 and *p* = 0.496) (Figure 5 and Supplementary Figure 2).

**Figure 6:**
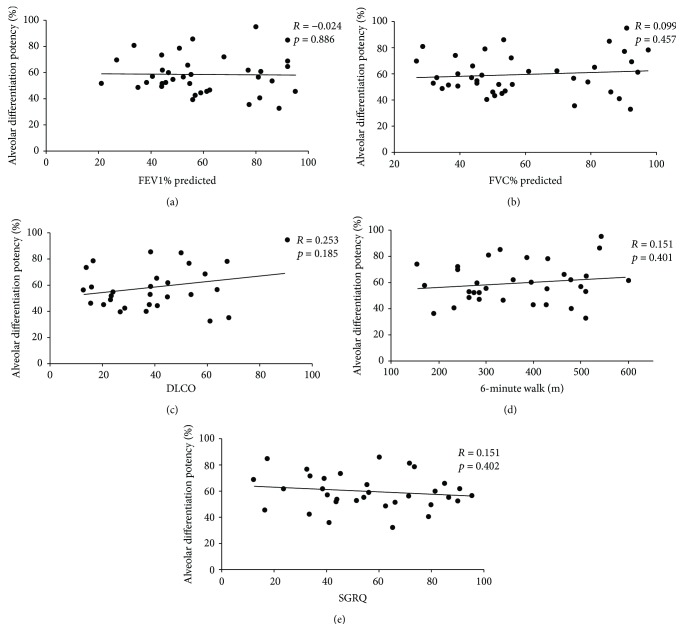
Correlation analysis between alveolar differentiation potency and lung function of the ILD patients. DASCs from ILD patients were examined by Pearson correlations to determine whether alveolar differentiation potency was related to measures of airflow (i.e., forced expiratory volume in one second (FEV1%) and forced vital capacity (FVC%)), diffusion (i.e., diffusing capacity of the lung for carbon monoxide (DLCO)), and quality of life (i.e., 6-minute walk and St. George's respiratory questionnaire (SGRQ)) in ILD patients.

To further examine whether alveolar differentiation potency was related to lung function, correlations were performed with measures of lung function that relate to the airflow (i.e., forced expiratory volume in one second (FEV1%) and forced vital capacity (FVC%)), diffusion (i.e., diffusing capacity of the lung for carbon monoxide (DLCO)), and quality of life (i.e., 6-minute walk and St. George's respiratory questionnaire (SGRQ)) in ILD patients. Consequently, alveolar differentiation potency rarely correlated with parameters of the tested lung function, with all correlation analyses failing to show significance (Figure 6). Similarly, no correlations of alveolar differentiation potency to lung function were observed in the bronchiectasis and COPD patients (Supplementary Figures 2 and 3). Altogether, the above results implied that the differentiation potency of DASCs from patients was less tightly associated with lung functions but was associated with the age of patients.

## 4. Discussion

It is known that in the unperturbed adult lung, the organ is relatively quiescent with remarkably slow cell turnover, especially when compared with other epithelial tissues, such as the skin and intestine [25]. However, after insult, injury, or infection, the lung has a tremendous potential to rapidly respond to the injury and replenish damaged tissue by switching from a homeostatic status to a regenerative status [26]. Numerous studies have suggested the existence of tissue-specific lung stem/progenitor cells with self-renewal and differentiation potential [27], although debates on definition and characterization of these stem/progenitor cell lineages still exist [28, 29]. The concept of active maintenance hypothesizing that there are signaling pathways that actively maintain lung quiescence is an emerging explanation for the tremendous regeneration capacity of the lung [30]. After injury, the quiescent signaling deactivates, leading to a transition of the putative lung stem cells from quiescence to proliferation and differentiation, and thus provides a rapid and robust generative process to repair the damaged tissue [31, 32]. Meanwhile, this is of importance for therapeutic application of lung regeneration since the reactivated pathways that maintain quiescence in the adult lung may provide an intrinsic brake and prevent uncontrollable cell division caused by endogenous or exogenous stem cells [33]. Therefore, given the specific regenerative features of the lung, the concept of introducing autologous stem/progenitor cells to the damaged lung tissue for treatment of lung diseases is quite attractive.

Previously, we have provided solid evidence demonstrating that a KRT5+P63+SOX9+ subpopulation of adult lung basal cells existing in the distal airway epithelium holds the ability to differentiate into multiple pulmonary cell types and could repopulate the lung epithelium after damage. The purpose of this study was to further confirm their therapeutic potential by directly assessing the differentiation capacity of DASCs isolated from a cohort of human subjects suffering from chronic degenerative lung diseases. By comparing the cell morphology and expression pattern of DASCs and TSCs after differentiation, we demonstrated that human basal stem cells originating from different anatomic regions of the airway may hold distinct lineage commitment, owing to the complex composition and organization of the respiratory epithelium [34]. More importantly, single-cell-derived clones showed diverse differentiation fates in the same culture system, even if the clones arise from the same person or different individuals.

Another highlight in the present study is that we provided a simple and convenient quantification method for measurement of the DASC differentiation ability by introducing a monolayer differentiation system in a serum-free condition. The maturation of AEC1s, reflected by the HOPX expression, should be taken into full consideration since AEC1s, specialized for gas exchange, cover more than 90% of the alveolar surface area and have been reported to have very limited proliferative capacity in vivo [35, 36]. This measurement can serve as a helpful parameter to determine the differentiation ability of the isolated lung stem cells, particularly under the circumstances when time is limited and simplified analysis is needed.

As an example, we differentiated DASCs sampled from 85 subjects and compared their alveolar differentiation potency, aiming to reveal the differentiation pattern of DASCs under pathological conditions. Of note, we found that the DASCs from patients showed significantly higher portion of AEC1 after differentiation compared to those from normal individuals. This is intriguing since several studies have reported an “exhaustion” and dysfunction of basal progenitors in COPD airways [17, 37]. Yet, inconsistency has emerged when the basal cells were subjected to an in vitro air-liquid interface (ALI) culture. Staudt et al. reported similar numbers of ciliated cells in an ALI culture shown by *β*-tubulin IV expression of nonsmoker, healthy smoker, and COPD smoker samples, which were due to DNA methylation change [37], while a study by Ghosh et al. observed the ciliated cell hypoplasia during ALI culture of COPD basal cells [17]. As we have shown here that in COPD patients, DASC showed increased type I alveolar differentiation potential, it seems that the lung tissue injury affects the differentiation bias of DASC and favors alveolar lineage.

Through further analysis regarding the correlation of DASC differentiation capacity with lung diseases, we noted a strong relationship of aging and DASC differentiation in bronchiectasis and ILD populations. The lung is considered as an “age-sensitive” organ since the incidence of most chronic lung diseases increases with age [38–40]. According to a study focusing on mouse trachea and main stem bronchi, senescence of the lung epithelium is associated with changes of the cellular composition, organization, and local microenvironment. The authors observed a decline in the number of Krt5+P63+ basal cells present in the trachea epithelium of old mice [41]. Consistently, our study further confirmed that the differentiation potential of lung basal stem cells also decreases with aging in human patients. A comprehensive understanding of this aspect may help to improve the prognosis for patients treated with cell-based therapies.

One of the limitations in the current study is that it is an exploratory clinical research with a relatively small number of subjects diagnosed with bronchiectasis and COPD. Additionally, only 6 healthy volunteers with normal spirometry were included in this cross-sectional study because of ethical reasons. Therefore, the correlation of DASC differentiation potency with age and lung diseases reported in this study must be cautiously interpreted. To verify our observation, more cases of bronchiectasis and COPD are required and independent analysis should be performed for each disease type. Another limitation is not having a description of the differentiation potency to AEC2s in the whole clinical cohort. It would have been beneficial to know the difference among patients regarding the frequency of AEC2s yielded in the monolayer differentiation system.

In summary, our data highlighted the therapeutic potential of human DASCs in patients suffering from bronchiectasis, COPD, and ILD. In general, a lineage diversity of DASC differentiation potential among individuals was noted by us, and this diversity is related to multiple factors including the anatomic region, age, and pathologic conditions of the individuals.

## Figures and Tables

**Table 1:** Baseline demographic and clinical characteristics of study subjects.

	Healthy control	BE	COPD	ILD
*N* subjects	6	13	21	45
Age (years)	45.3 ± 10.76	42.6 ± 9.22	60.1 ± 9.92	58.3 ± 9.74
Male/female	3/3	5/8	20/1	34/11
Body mass index	22.6 ± 4.08	21.7 ± 3.57	21.5 ± 3.16	24.4 ± 4.25
Disease duration (years)	NA	12.0 ± 7.85	11.4 ± 9.64	4.6 ± 5.20^a^
FEV1% of predicted value	NA	50.3 ± 21.38	33.5 ± 17.62	60.0 ± 20.32^a^
FEV1% of FVC2	NA	65.8 ± 16.09	43.3 ± 9.52	86.0 ± 12.39^a^
FVC% of predicted value	NA	66.8 ± 20.35	57.9 ± 17.87	59.4 ± 21.84^a^
DLCO% of predicted value	NA	68.8 ± 19.93	38.2 ± 17.67	39.7 ± 19.51^b^
6-minute walk (meters)	NA	362.1 ± 119.5	406.5 ± 112.4	367.2 ± 121.1^c^
SGRQ	NA	44.3 ± 27.94	45.7 ± 15.19	55.5 ± 23.38^c^

^a^Data from 37 patients were collected. ^b^Data from 29 patients were collected. ^c^Data from 33 patients were collected.

## Data Availability

The data used to support the findings of this study are available from the corresponding author upon request.

## Supplementary Materials

Supplementary Materials Supplementary Figure 1: alveolar differentiation of DASCs generated from bronchiectasis, COPD, ILD, and normal lungs. Supplementary Figure 2: the correlation between gender and alveolar differentiation potency of DASCs. Supplementary Figure 3: correlation analysis between alveolar differentiation potency and lung function of the bronchiectasis patients. Supplementary Figure 4: correlation analysis between alveolar differentiation potency and lung function of the COPD patients.
