# Supplementary material for: Alveolar Differentiation Potency of Human Distal Airway Stem Cells Is Associated with Pulmonary Pathological Conditions
Source: Stem Cells Int. 2019 Jun 9;2019:7123078. doi: 10.1155/2019/7123078 (PMC6590602; doi:10.1155/2019/7123078)
Supplement: Supplementary Materials — Supplementary Figure 1: alveolar differentiation of DASCs generated from bronchiectasis, COPD, ILD, and normal lungs. Supplementary Figure 2: the correlation between gender and alveolar differentiation potency of DASCs. Supplementary Figure 3: correlation analysis between alveolar differentiation potency and lung function of the bronchiectasis patients. Supplementary Figure 4: correlation analysis between alveolar differentiation potency and lung function of the COPD patients. [file 7123078.f1.pdf]

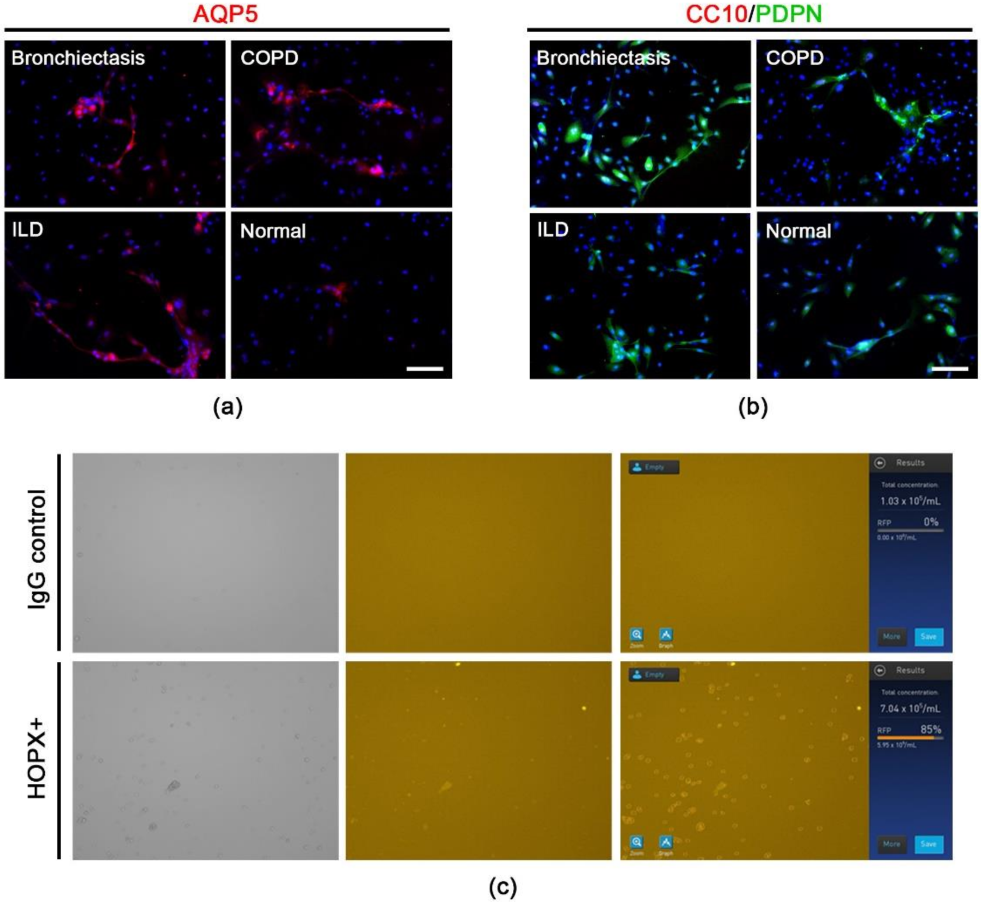

Supplementary Figure 1: alveolar differentiation of DASCs generated from bronchiectasis, COPD, ILD and normal lungs. (a) Immunostaining of AQP5 expression on monolayer differentiated DASCs from bronchiectasis, COPD, ILD and normal lungs. Scale bar, 50 μm. (b) Co-staining of CC10 and PDPN on monolayer differentiated DASCs from bronchiectasis, COPD, ILD and normal lungs. Scale bar, 50 μm. (c) A representative example showing how the HOPX+ cell frequency was determined by a fluorescent cell counter. Briefly, total cells were counted under bright field (left) and then cells with positive fluorescence were counted by using a fluorescent filter(middle). Finally, positive cell frequency was calculated(right). The IgG control sample was used to set the bottom-line of the fluorescent signals. Similar gating strategies were used to ensure the consistency of the measurement.

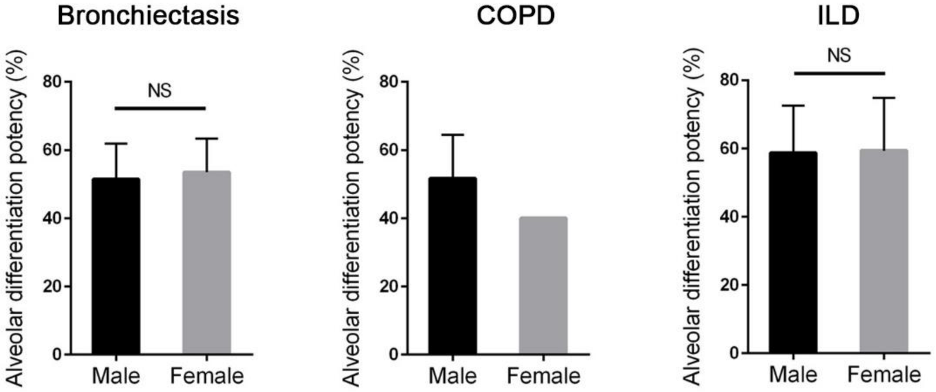

Supplementary Figure 2: The correlation between gender and alveolar differentiation potency of DASCs. NS, not significant.

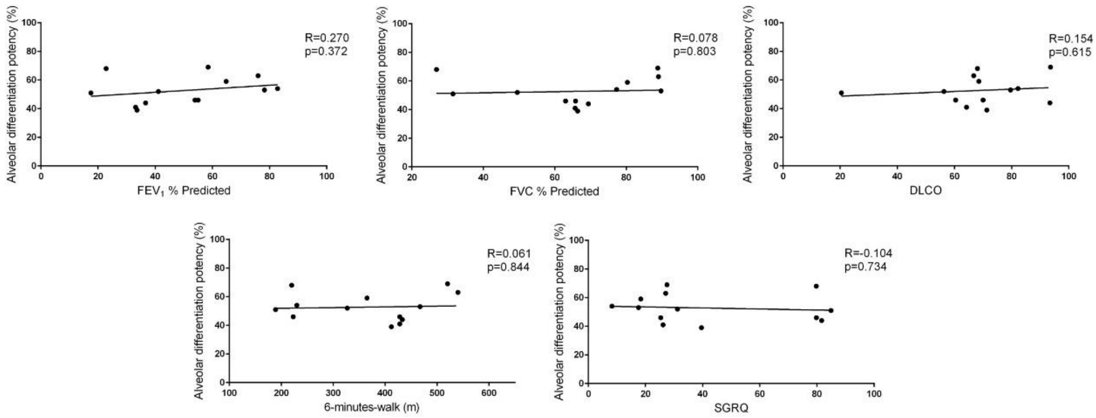

Supplementary Figure 3: Correlation analysis between alveolar differentiation potency and lung function of the bronchiectasis patients. DASCs from bronchiectasis patients were examined by Pearson correlations to determine whether alveolar differentiation potency were related to measures of airflow (i.e. forced expiratory volume in one second (FEV1%) and forced vital capacity (FVC%)), diffusion (i.e. diffusing capacity of the lung for carbon monoxide (DLCO)) and quality of life (i.e. 6-minutes-walk and St George’s respiratory questionnaire (SGRQ)) in ILD patients.

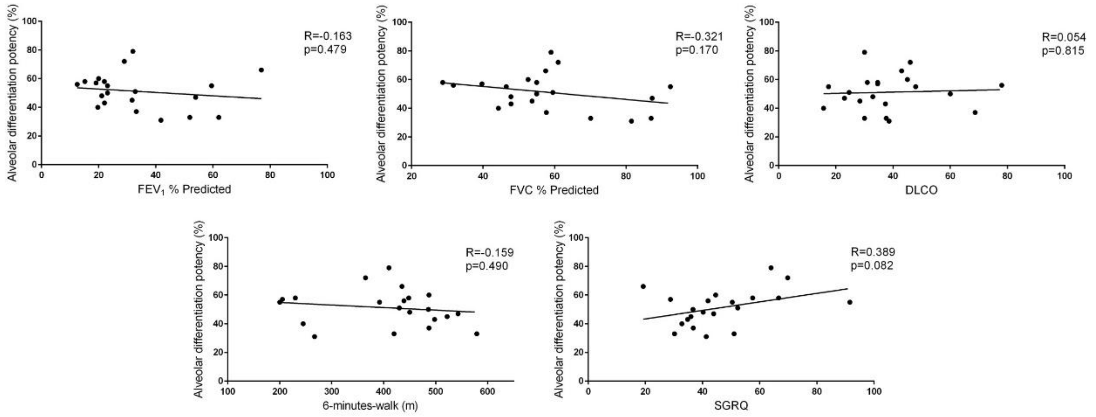

Supplementary Figure 4: Correlation analysis between alveolar differentiation potency and lung function of the COPD patients. DASCs from COPD patients were examined by Pearson correlations to determine whether alveolar differentiation potency were related to measures of airflow (i.e. forced expiratory volume in one second (FEV1%) and forced vital capacity (FVC%)), diffusion (i.e. diffusing capacity of the lung for carbon monoxide (DLCO)) and quality of life (i.e. 6-minutes-walk and St George's respiratory questionnaire (SGRQ)) in COPD patients.
